# Physiological Responses of Earthworm Under Acid Rain Stress

**DOI:** 10.3390/ijerph17197246

**Published:** 2020-10-03

**Authors:** Xuan Chen, Jiaen Zhang, Hui Wei

**Affiliations:** 1 College of Natural Resources and Environment, South China Agricultural University, Guangzhou 510642, China; xuanchen@stu.scau.edu.cn (X.C.); weihui@scau.edu.cn (H.W.); 2 Guangdong Provincial Key Laboratory of Eco–Circular Agriculture, Guangzhou 510642, China; 3 Guangdong Engineering Research Center for Modern Eco-agriculture and Circular Agriculture, Guangzhou 510642, China; 4 Key Laboratory of Agro-Environment in the Tropics, Ministry of Agriculture and Rural Affairs, Guangzhou 510642, China

**Keywords:** earthworm, acid rain, physiological responses, CYP3A4, epidermal damage

## Abstract

Acid rain has become one of the major global environmental problems, and some researches reported that acid rain may have a certain inhibition on soil biodiversity. Besides this, it is well known that earthworm (*Eisenia fetida*) plays an important role in the functioning of soil ecosystems. For this point, we conducted a series of experiments to investigate whether acid rain would take effects on earthworms. In the present study, the earthworms were incubated on filter paper and in soil under acid rain stress. The mortality and behavior of earthworms were recorded, and epidermal damage and the activity of the CYP3A4 enzyme were measured for the tested earthworms. Our experimental results showed that the earthworms could not survive in the acid rain stress of pH below 2.5, and acid rain with weak acidity (i.e., 4.0 ≤ pH ≤ 5.5) promoted the activity of the CYP3A4 enzyme in the earthworms, while acid rain with strong acidity (i.e., 3.0 ≤ pH ≤ 3.5) inhibited it. Moreover, the degree of damage in sensitive parts of the earthworms increased with the decrease of pH value. This study suggests that acid rain can cause discomfort response and the direct epidermal damage of earthworms, and even kill them.

## 1. Introduction

In the last decades of the twentieth century, acid rain was identified as one of the most serious environmental problems [1]. For some time, especially in the 1980s, many people thought that acid rain was one of the most serious environmental threats [2]. During that time, through the news media reports, people learned about the degradation of forests on the European Continent and the extinction of fish in Scandinavian surface waters. Not only that, even in North America, people understood the harm of acid rain to the environment. Therefore, acid rain has received more and more attention from the public. Today, acid rain has expanded from industrialized countries to developing countries [3] and has gradually evolved into a worldwide problem [4].

Acid rain is the result of the rapid development of industry and commerce [5,6], and more and more studies have paid attention to the impact of acid rain on ecosystems. For instance, the results of Zhang et al. [7] showed that acid rain at pH 3.5 and pH 2.5 inhibited photosynthesis and antioxidant defense system of tea tree, causing metabolic disorder, and ultimately affected the growth and development of the plants, while the acid rain at pH 4.5 had no toxic effects on tea seedlings. Jin et al. [8] showed that acid rain could significantly reduce the carbon, nitrogen, and phosphorus contents of early rice leaves at the jointing stage (*p* < 0.05). Also, Chen et al. [9] indicated that in the whole growing season of early rice, the effect of acid rain on total organic carbon (TOC) content of the soil was not significant (*p* > 0.05). However, in the green and mature period of late rice, the acid rain significantly increased TOC content (*p* < 0.05). Moreover, the content of soil microbial biomass carbon (MBC) in early rice and late rice showed a significant change with time, which showed that the content of MBC in the early rice was lower than those in the acid rain treatment (*p* < 0.05), while for those in the late rice, it was the opposite. Other studies have focused on soil microbes, and there has been a lot of work on the function and structure of litter-decomposing microbial communities under acid rain [10]. In the study, the results presented that the types of litter affected the functions and community composition of microbes, but there was non-significant difference between acid rain treatments. Furthermore, Liu et al. [11] showed that both acid rain of pH 4.0 and pH 3.0 significantly increased the total amount of soil microbial phospholipid fatty acids (PLFAs) by increasing the PLFA contents of gram-positive bacteria, actinomycetes, fungi, and arbuscular mycorrhizal fungi. Most of the existing studies have focused on the impact of acid rain on plants, soil nutrients, and soil microorganisms, but few have focused on the impact of acid rain on soil animals, especially on earthworms. Even though soil animals make only a small direct contribution to the energy flow in terrestrial ecosystems [12], they have a strong indirect effect on energy flow due to the strong influence of litter fragmentation, inoculation, bioturbation, and soil aeration on microbial mineralization and immobilization processes [13,14]. Earthworm is one of the most important invertebrates in terrestrial ecosystems [15], it plays not only an irreplaceable role in maintaining the function of the soil ecosystem but also an important bridge to communicate the connection between soil and terrestrial organisms through the food chain [16]. Earthworms have an enrichment effect on most of the pollutants entering the soil [17] and have been recommended by international organizations such as Organization for Economic Co-operation and Development (OECD) and International Organization for Standardization (ISO) as important indicator organisms for ecological risk assessment of toxic substances in terrestrial environments [18,19,20]. Recently, some studies have shown that soil pollution [21,22,23] and acid rain [24,25] are severe in Southern China. Therefore, it is of great significance to investigate whether and how acid rain will influence earthworms in this area. The previous study showed that, due to acid toxicity, earthworms tried to stay away from the soil and died due to acid rain at pH = 2.0, and the activity of catalase in the earthworms decreased with the increase of pH value of acid rain, while the activity of superoxide dismutase showed a fluctuating pattern [26]. Therefore, this study will further evaluate the possible harm of acid rain to earthworms. The aim is to explain the toxic effects of acid rain on earthworms through external morphological changes and internal enzyme changes.

Certain pollutants in the environment can induce or inhibit CYP (Cytochrome P450), resulting in changes in enzyme activity, which in turn affects the metabolism of itself and other substances. CYP3A4 is one of the most important sub-enzymes in CYP450, and it is involved in the metabolism of more than 150 drugs (about 50% of all drugs) in about 38 categories [27]. In particular, it has a special indicative effect on endocrine disruptors [28,29,30]. Therefore, the activity of the CYP3A4 enzyme can be used as a potentially sensitive biomarker for the toxicological diagnosis of pollutants in soil. Besides this, it is of great significance to accurately evaluate the response relationship between CYP3A4 enzyme activity and pollutants for the ecotoxicological study of environmental pollution.

In this study, based on the situation of acid rain in Guangzhou, China, we investigated the effect of acid rain on *Eisenia fetida*. The short-term filter paper experiment (72 h) and soil experiment (15 days) were used to clarify the physiological responses of the earthworms, and the activity of the CYP3A4 enzyme in the earthworms under acid rain stress (with a pH of 2.0, 2.5, 3.0, 3.5, 4.0, 4.5, 5.0, 5.5, 7.0) was studied. We hypothesized that acid rain would cause adverse effects on the earthworms by damaging the epidermis and changing physiological characteristics, such as the activity of the CYP3A4 enzyme. This study would provide a reference for clarifying the physiological and ecological changes of soil organisms in response to acid rain stress.

## 2. Materials and Methods

### 2.1. Experimental Device

**Figure 1:**
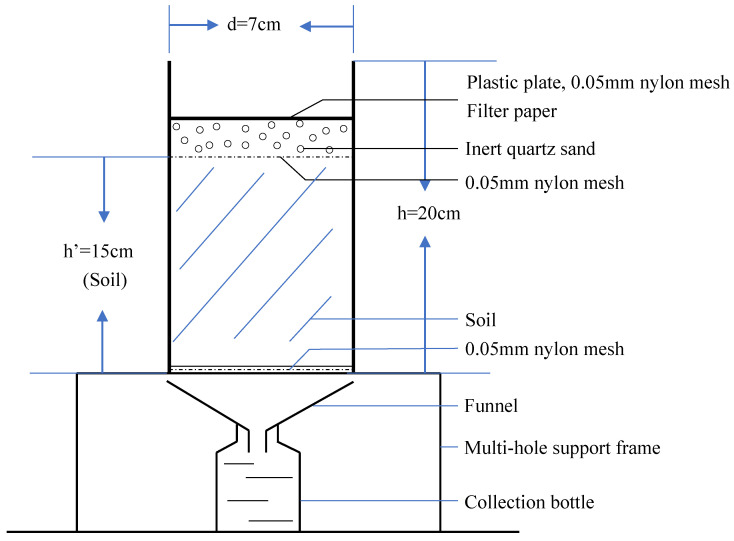
Schematic diagram of the constructed soil column.

The soil column for the test was divided into two parts. The upper part was the *leaching device* and the lower part was the *collection device*. The *leaching device* was made of PVC pipe (d = 7 cm, h = 20 cm), and the filling order from top to bottom was plastic plate, 0.05 mm nylon mesh, filter paper, inert quartz sand with a thickness of about 10 mm, 0.05 mm aperture nylon mesh, soil with a height of about 15 cm, and 0.05 mm nylon mesh (Figure 1). Nylon mesh and quartz sand were used to make the leaching as uniform as possible. The entire *leaching device* was placed on a multi-hole support frame until the end of the experiment. The top-down order of the *collection device* was funnel and brown glass bottle and placed in the multi-hole support frame.

### 2.2. Tested Materials

#### 2.2.1. Preparation of Acid Rain

According to the characteristics of acid rain in Guangzhou, mother liquor (pH = 1.0) for the simulated acid rain was prepared with the molar ratio of sulfate to nitrate at 5:1 [31], and it was sealed and stored for reserve. Then, the mother liquor was used to dilute into leaching solutions with pH values of 2.0, 2.5, 3.0, 3.5, 4.0, 4.5, 5.0, and 5.5, respectively. Deionized water (pH = 7.0) was set as the control. In this experiment, as standard catalog items, analytical grade sulfuric acid (H_2_SO_4_) and nitric acid (HNO_3_) were purchased from Guangzhou Chemical Reagent Manufacture Inc., China.

#### 2.2.2. Tested Earthworm

The earthworm used in the experiment was *Eisenia fetida*, provided by the Department of Soil Science, College of Natural Resources and Environment, South China Agricultural University, China. Healthy adult earthworms with clitella were selected at the age of 2 to 3 months, with a weight of 200 to 500 mg. Before the experiment, the earthworms were pre-cultured in clean soil for 7 days to adapt to the tested soil environment.

#### 2.2.3. Tested Soil

The experimental latosol was taken to a depth of 20 cm from a forest garden located on the campus of South China Agricultural University, Guangdong Province, China. In this work, the air-dried soil was crushed and sieved after passing through the sieve of 2-mm mesh, which had a pH of 4.3, an organic matter content of 44.87 g·kg^−1^, a base saturation of 13.1%, and a cation exchange capacity of 6.70 mmol·kg^−1^. Furthermore, the initial contents of soil cation, including H^+^, Ca^2+^, Na^+^, Al^3+^, K^+^, and Mg^2+^, were 0.378, 0.781, 0.048, 3.211, 0.072, and 0.0683 cmol·kg^−1^, respectively.

### 2.3. Experimental Design

#### 2.3.1. Exposure Experiments

In this study, the earthworms were incubated on filter paper and in soil under acid rain stress.

In the filter paper test, the bottom diameter of the beaker was 2.4 cm and the height was 15 cm. A layer of filter paper was put at the bottom of the beaker, the earthworms were placed on the paper, then 3 mL of acid rain was applied to them. The beaker was sealed with a plastic film with pinholes and then placed in an incubator (Hadonglian HPG-280BX) at 20 ± 2 degrees ℃ to avoid light. This test consisted of 9 treatments, which refer to different pH values respectively, each treatment had 10 replications and each repetition contained an earthworm. Furthermore, the entire experiment lasted 72 h.

In the soil test, 0.5 kg of air-dried soil was filled into each soil column, and the soil columns were placed in pure water for 48 h, then removed and stood still for 72 h. Based on the average annual precipitation in Guangdong in 2018 (1843 mm), 30% of the annual precipitation was selected as the surface runoff [32], and the total leaching volume was determined to be 4.97 L. In this study, an intermittent leaching method was used, and 994 mL simulated acid rain leached into the column at one time. Controlling the flow rate with a peristaltic pump of 0.35 mL·min^-1^, leaching every 72 h, a total of 5 times. Therefore, the cumulative leaching amount was equivalent to the local precipitation in 2018. In the test, 10 earthworms treated with jejunum for 24 h were added to each soil column and cultured in an incubator (Hadonglian HPG-280BX) at 20 ± 2 degrees °C; for 15 days. This test involved 9 treatments which refer to different pHs and each pH treatment was tested in 3 replications with 10 earthworms per soil column. The pathological symptoms and behaviors of the earthworms in two culture experiments were observed and recorded daily, including morphological change, swelling, and activity. When there was no response to the stimulation on the earthworm’s tail, this result indicated that the earthworm was dead.

#### 2.3.2. Measurement of CYP3A4 Enzyme Activity

After the earthworm was disintegrated, the tissue was extracted. Then the microsomal protein was prepared and incubated in vitro. Using testosterone (Sigma Aldrich (Shanghai) Trading Co., Ltd., Shanghai, China) as probe drug and the activity of the CYP3A4 enzyme was determined by HPLC (Waters 2695) with methanol (Guangzhou Congyuan Instrument Ltd., Guangzhou China)/ultra-pure water (50/50, V:V) as the mobile phase. The detection wavelength was 254 nm and the flow rate was 1.5 mL·min^-1^. Furthermore, the retention time of 6β-hydroxytestosterone was 2 min [33,34,35].

#### 2.3.3. Scanning of the Electron Microscope of Earthworm Epidermis

At first, the samples were fixed with glutaraldehyde Fixation for 8 h. Secondly, they were soaked in 50% ethanol, 70% ethanol, 85% ethanol, 95% ethanol, and 100% ethanol respectively for 14 min, 14 min, 14 min, 15 min, and 15 min. Glutaraldehyde and ethanol were purchased from Guangzhou Congyuan Instrument Ltd., China. Thirdly, we freeze-dried the above treated samples for 3 h, then used double-sided conductive glue to stick the samples on the observation platforms and sprayed gold. Finally, we observed and took photos of the samples under the scanning electron microscope (Hitachi S-3400N) (HITACHI, Tokyo, Japan) [36].

### 2.4. Statistical Analysis

At first, the pathological symptoms and behavior of earthworms were recorded in a report on the number of observed effects. Second, Microsoft Office Excel 2007 (Microsoft Corporation, Redmond, WA, USA) was used for the data collection of the activity of the CYP3A4 enzyme, and the results were shown as means ± standard error. Third, SPSS 17.0 (IBM, Chicago, IL, USA) was used for statistical analysis, and all the data of the activity of the CYP3A4 enzyme were compared by Duncan’s method for significance at a significance level of 5%. Fourth, Origin 8.0 (OriginLab, Northampton, MA, USA) was used for graphing.

## 3. Results and Discussion

### 3.1. Behavior and Mortality of Earthworms

**Figure 2:**
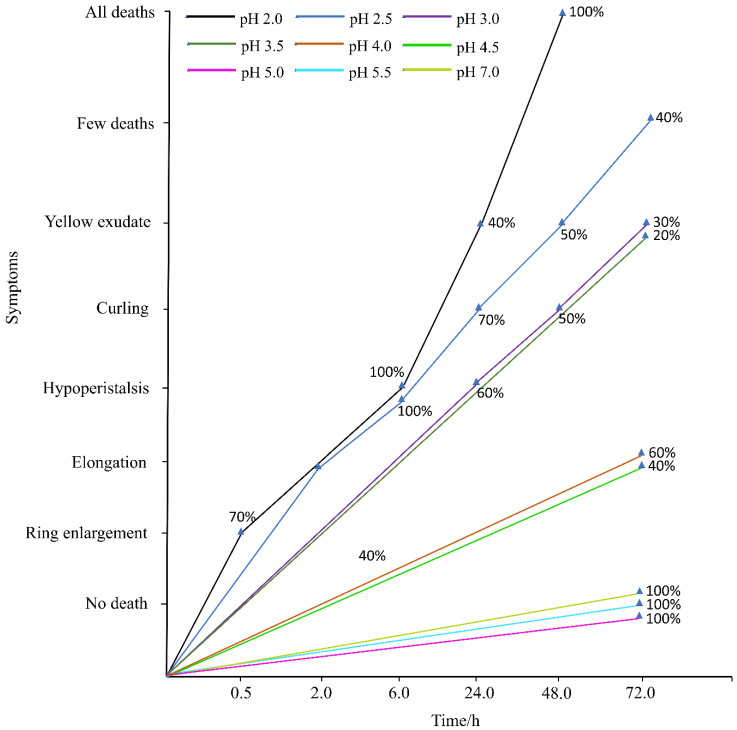
Behavior of adult *Eisenia fetida* on filter paper under acid rain with eight pH levels for 72 h (*n* = 10). Treatments: pH 7.0: control, pH 2.0–5.5: acid rain with different pH values. Percentages indicate the number of earthworms with the symptom.

Compared with the control (Figure 2), when exposed for 0.5 h in the filter paper experiments, the earthworms at pH 2.0 showed ring swelling and a sugar gourd shape. After 6 h, the earthworms at pH below 3.0 became sluggish. After 24 h, the earthworms at pH 2.0 appeared broken nodes, and the bodies were soft and eroded from the ring also with exuding yellow liquid, and 40% of the earthworms died. At the same time, 70% of the earthworms at pH 2.5 showed symptoms of poisoning with curling and bending. After 48 h, all the earthworms died at pH 2.0, and at pH 2.5, 50% of the earthworms exuded yellow liquid. After 72 h, the earthworms at pH 2.5 appeared broken nodes, and their bodies became soft and eroded from the ring, and 40% of the earthworms died. Meanwhile, 30% of the earthworms at pH 3.0 and 20% of the earthworms at pH 3.5 had yellow liquid exudation. Besides this, 60% and 40% of the earthworms elongated at pH 4.0 and pH 4.5, respectively. During the whole experiment period, the earthworms at pH 5.0 and pH 5.5 did not show obvious symptoms of acid rain poisoning. At the end of the experiment, the mortality of the earthworms in the treatment group with pH 2.0 was 100%, and that at pH 2.5 was 40% (Table 1). There was no death appeared in the other groups.

**Figure 3:**
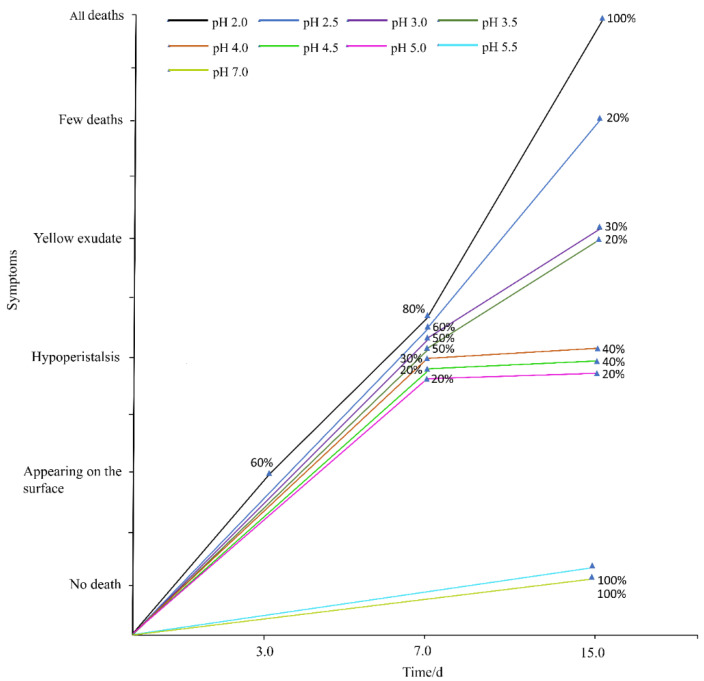
Behavior of adult *Eisenia fetida* in soil under acid rain with eight pH levels for 15 days (*n* = 10). Treatments: pH 7.0: control, pH 2.0–5.5: acid rain with different pH values. Percentages indicate the number of earthworms with the symptom.

The results of the soil test were consistent with those of the filter paper test (Figure 3). During the 15-day test period, the earthworms between pH 5.0 and 5.5 survived well without symptoms of acid rain poisoning. Compared with the control, 60% of the earthworms at pH 2.0 appeared the symptoms on the surface of the soil and tended to escape from the soil columns on the third day. After 7 days of leaching experiments, the earthworms in the treatment group with pH less than 5.0 wriggled slowly. After 15 days of acid rain treatment, the earthworms between pH 2.0 and pH 3.5 exuded yellow liquid and all the earthworms at pH 2.0 died on the surface of the soil. Furthermore, the surface of earthworms at pH 2.5 lost luster, the color became dark, and 20% of them died. In this experiment, the mortality of the earthworms in the acid rain treatment at pH 2.0 was 100%, and that at pH 2.5 was 20% (Table 2). But there was no death in the other groups.

The effects of acid rain on soil animals can be divided into the following two categories. One is that the death caused to soil animals in contact with or deep into the body is called direct impact [35,37]. The other is that the pH value of the soil is reduced due to the falling and adsorption of acid rain, which is called indirect effect [38]. In the death caused by direct contact and entry into the body, sensitive mammals and weakly resistant animals are most affected by acid rain, including nematodes and earthworms [39,40]. The filter paper experiments in this study indicated that earthworm was susceptible to acid rain under the experimental conditions. Studies have shown that the adverse effect of acid rain on the growth of earthworms increases with the decrease of pH value of acid rain [41], which may be that H^+^ in acid rain reduces the pH value of cells and changes the normal pH required for life activities such as earthworm growth, development, and reproduction [42].

When acid rain enters into the soil, on one hand, SO_4_^2−^ and NO^3−^ in acid rain cause the decrease of soil pH and salt-based saturation, while carrying a certain amount of cations away from the soil, resulting in the leaching of salt-based ions (K^+^, Na^+^, Ca^2+^, Mg^2+^, NH^4+^), which exacerbates the process of soil acidification and thus affects the activity of earthworms [43,44]. On the other hand, acid rain promotes the release of heavy metals in soil and improves its bioavailability [44]. Although earthworms have a certain tolerance limit to heavy metals when the content of heavy metals exceeds the limit, heavy metals will produce toxic effects, and then affect the normal physiological function of earthworms [45]. Reinecke et al. [46] found that Pb-contamination had no significant effect on the cocoon production rate of earthworms, but had a significant impact on its viability. Salminen et al. [47] studied the resistance of *Cognettia sphagnetorum* to Cu and found that the sizes of adults were smaller in the polluted plots. Furthermore, heavy metal contamination can damage the microstructure of cells, make the activity of enzymes abnormal, and affect the repair ability of DNA. Guo et al. [48] and Wang et al. [49] also reported that heavy metals could cause severe damage to the epidermis, gastrointestinal tract, and cell microstructure of earthworm.

### 3.2. The Epidermal Damage of Earthworms

**Figure 4:**
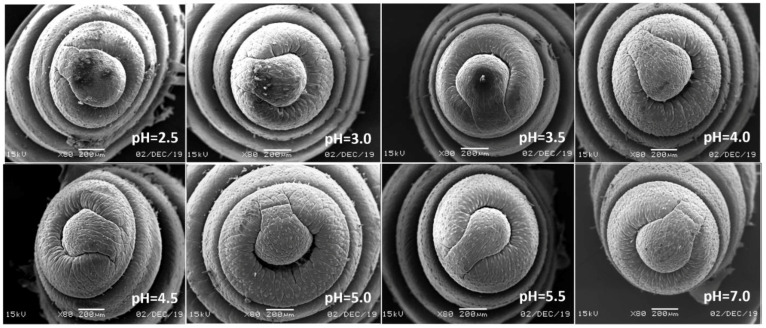
Prostomium of adult *Eisenia fetida* on filter paper under acid rain with eight pH levels for 72 h (*n* = 10). Treatments: pH 7.0: control, pH 2.0–5.5: acid rain with different pH levels.

**Figure 5:**
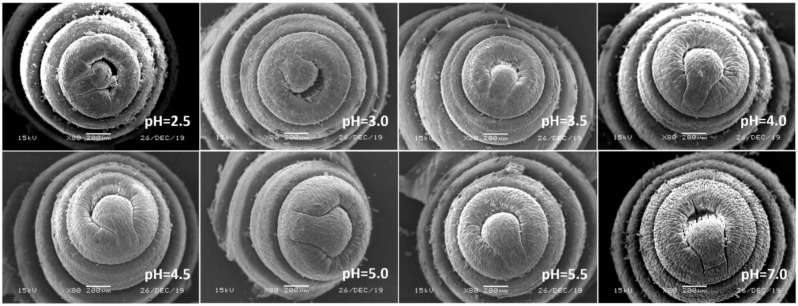
Prostomium of adult *Eisenia fetida* in soil under acid rain with eight pH levels for 15 days (*n* = 10). Treatments: pH 7.0: control, pH 2.0–5.5: acid rain with different pH levels.

**Figure 6:**
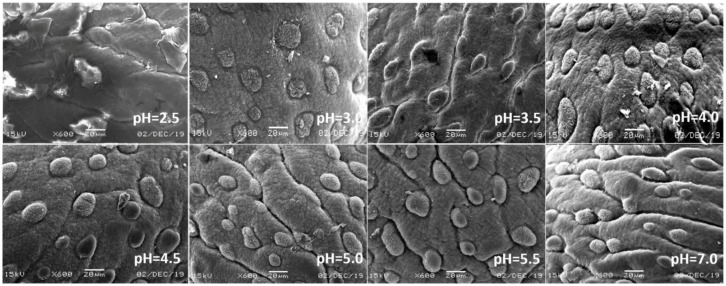
Epidermis of the first segment of adult *Eisenia fetida* on filter paper under acid rain with eight pH levels for 72 h (*n* = 10). Treatments: pH 7.0: control, pH 2.0–5.5: acid rain with different pH levels.

**Figure 7:**
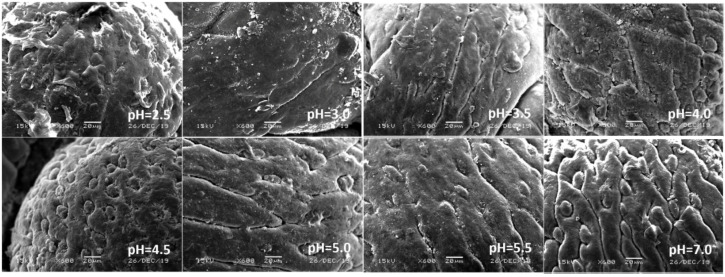
Epidermis of the first segment of adult *Eisenia fetida* in soil under acid rain with eight pH levels for 15 days (*n* = 10). Treatments: pH 7.0: control, pH 2.0–5.5: acid rain with different pH levels.

**Figure 8:**
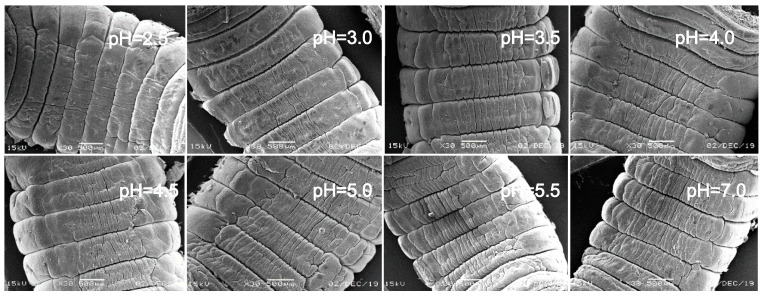
Ventral surface of the reproductive ring of adult *Eisenia fetida* on filter paper under acid rain with eight pH levels for 72 h (*n* = 10). Treatments: pH 7.0: control, pH 2.0–5.5: acid rain with different pH levels.

**Figure 9:**
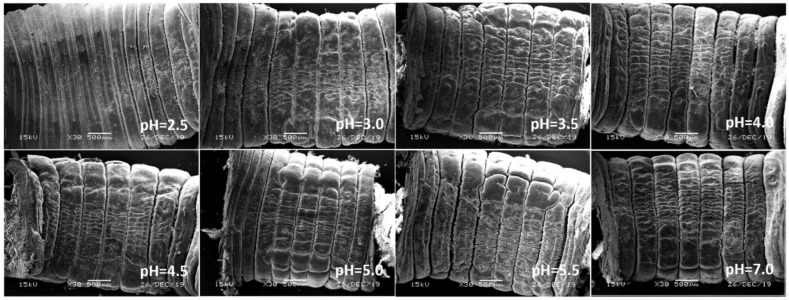
Ventral surface of the reproductive ring of adult *Eisenia fetida* in soil under acid rain with eight pH levels for 15 days (*n* = 10). Treatments: pH 7.0: control, pH 2.0–5.5: acid rain with different pH levels.

**Figure 10:**
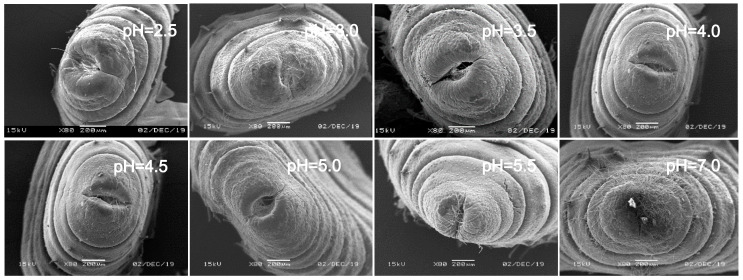
Anus of adult *Eisenia fetida* on filter paper under acid rain with eight pH levels for 72 h (*n* = 10). Treatments: pH 7.0: control, pH 2.0–5.5: acid rain with different pH levels.

**Figure 11:**
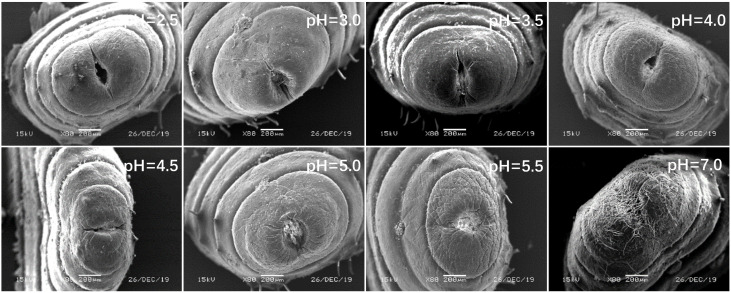
Anus of adult *Eisenia fetida* in soil under acid rain with eight pH levels for 15 days (*n* = 10). Treatments: pH 7.0: control, pH 2.0–5.5: acid rain with different pH levels.

In view of the above point regarding the damage of earthworms, the sensitive parts of earthworms (prostomium, ventral surface of reproductive ring, anus) were scanned by an electron microscope. The results showed that in the control, the texture of the prostomium of the earthworms was clear, the epidermis was smooth and elastic, the ventral surface of reproductive rings was clear and evenly distributed, and anal hair was attached to the anus. In the present study, we found that the results of filter paper experiments and soil experiments were consistent (Figure 4, Figure 5, Figure 6, Figure 7, Figure 8, Figure 9, Figure 10 and Figure 11). That is, the degree of damage in sensitive parts (prostomium, epidermis of the first segment, ventral surface of reproductive ring, anus) of earthworms in both culture experiments increased with the decrease of treatment pH. When pH ≤ 5.5, the anal hairs of earthworms in both cultures decreased significantly, and the stria on the epidermis of the first segment of earthworms in the soil disappeared obviously. The same symptoms also occurred in the filter paper experiments when pH ≤ 4.5. When pH ≤ 4.0, the anus of earthworms swelled in both culture experiments. In addition, when pH ≤ 3.5, ulcerous foci appeared in the prostomium of earthworms on the filter paper and in the anus of earthworms in the soil. Besides this, the texture on the ventral surface of the reproductive ring of the earthworms in the two cultures was blurred by swelling only in extremely strong acid rain (i.e., pH = 2.5).

Li et al. [50] reported that the cells would swell and thicken with water at acidic pH, the contact area and volume would increase, and even the cell membrane would be broken under the strong acidic condition. This is consistent with the results in this study, so it may be one of the reasons for the occurrence of ulcerous foci in earthworms. Other studies suggested that acid rain increases the risk of heavy metal contamination in soil [43]. Wan et al. [3] studied the effects of simulated acid rain on the release of aluminum ion in the soil and the results showed that the pH of the solution affected the aluminum content in the soil leaching solution, and with the decrease of the acid rain pH, the aluminum content in the leaching solution increased. In addition, the longer the bath time of the same pH solution, the greater the concentration of aluminum ion in the leaching solution. Another study suggested that when soil pH was less than 5.0, the dissolution concentration of active aluminum increased significantly and sharply with the decrease of pH [51]. When heavy metals in soil reach a certain amount, it can cause ulcers and tumors on the surface of earthworms [48].

### 3.3. CYP3A4 Enzyme Activity in Earthworms

Based on the external performances of earthworms under acid rain stress, we further studied the activity of the CYP3A4 enzyme of the treated earthworms. Cytochrome P4503A4 enzyme (i.e., CYP3A4) is an important enzyme that mainly exists in liver and small intestine. It can oxidize exogenous organic small molecules (heterotypic biomass), such as toxins or drugs so that they can be discharged from the body of earthworm.

**Figure 12:**
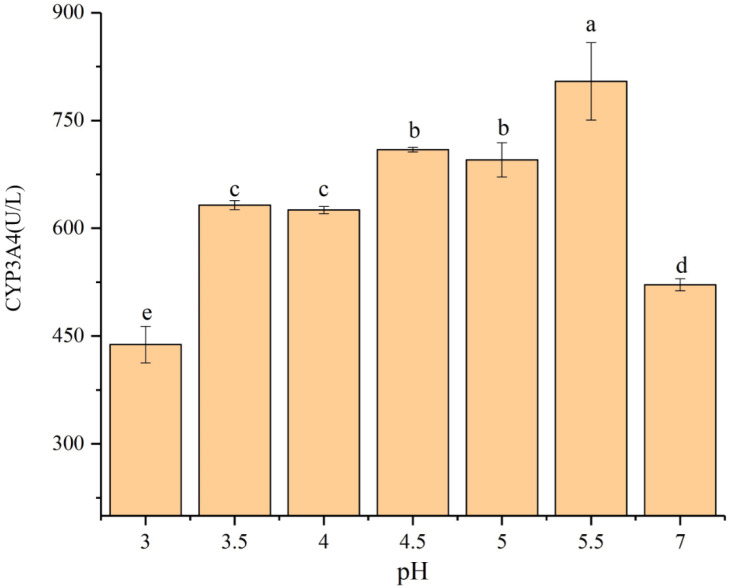
CYP3A4 enzyme activity in adult *Eisenia fetida* exposed to control filter paper and acid rain contaminated filter paper with eight pH levels for 72 h (*n* = 10). Error bars indicate standard error of the means (*n* = 10). Different letters indicate significant difference among treatments (*p* < 0.05).

**Figure 13:**
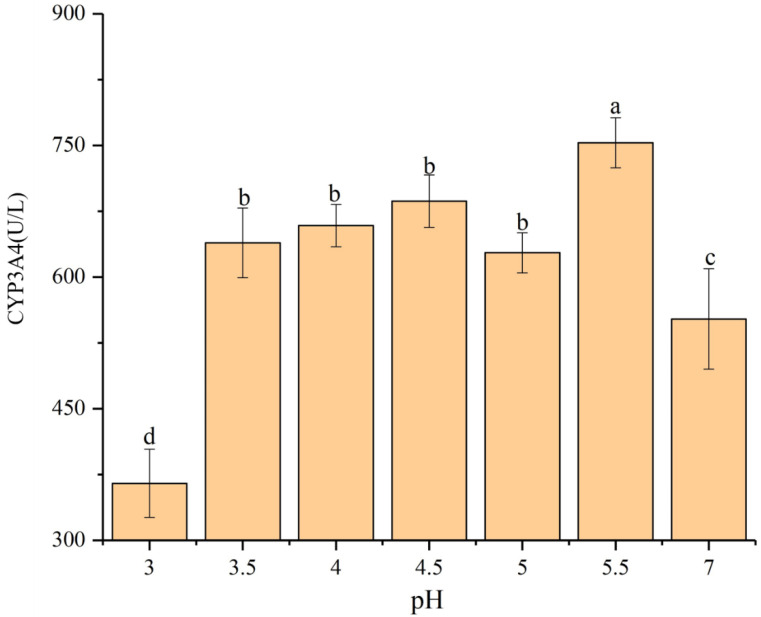
CYP3A4 enzyme activity in adult *Eisenia fetida* exposed to control soil and acid rain contaminated soil with eight pH levels for 15 days (*n* = 10). Error bars indicate standard error of the means (*n* = 10). Different letters indicate significant difference among treatments (*p* < 0.05).

In this study, in the filter paper experiments, relative to the control, acid rain increased the activity of the CYP3A4 enzyme in the earthworms by 21.3% (pH 3.5), 19.9% (pH 4.0), 36.1% (pH 4.5), 33.3% (pH 5.0), and 54.3% (pH 5.5), respectively (*p* < 0.05) (Figure 12), and significantly reduced it by 19.0% at pH 3.0 (*p* < 0.05). Furthermore, in the soil test, the activity of the CYP3A4 enzyme in the earthworms was also increased by 15.7% (pH 3.5), 19.3% (pH 4.0), 24.3% (pH 4.5), 13.7% (pH 5.0), and 36.4% (pH 5.5), respectively (*p* < 0.05) (Figure 13). Meanwhile, at pH 3.0, acid rain had an inhibitory effect on the CYP3A4 enzyme activity and reduced by 33.9% (*p* < 0.05).

As can be seen from the results, in a short period, in medium-intensity acid rain treatments (i.e., 3.5 ≤ pH ≤ 5.5), the activity of the CYP3A4 enzyme in the earthworms was significantly increased, indicating that acid rain induced its large-scale expression in the earthworms, which proves that the first-phase metabolic enzyme P450 played an important role in metabolism under acid rain stress [36].

Studies have shown that the change of pH can affect the degree of dissociation of certain groups on the active center of the enzyme, as well as the degree of dissociation of the substrates and coenzymes, thereby affecting the combination and catalysis of the enzyme molecules to the substrate molecules [52]. When the pH is too low, the enzyme protein will be inactivated due to denaturation [53]. Wang [54] showed that when pH < 5.0, CYP102A16 enzyme activity was low and less than 15% of the enzyme activity under the optimal pH condition. In this experiment, although the activity of the CYP3A4 enzyme in the earthworms at pH 3 in both culture methods was reduced compared with the control, the difference between the soil test and the control was more significant (*p* < 0.05), which may be because, in the filter paper test, pH is the only factor that affects the activity of the CYP3A4 enzyme, while in the soil test, the soil is a complex ecosystem. When acid rain enters the soil, it can not only directly affect the pH of soil [55], but also can cause the leaching of salt-based ions, and acid rain with strong acidity can accelerate this process [56]. A previous study has confirmed that excessive salt-based ions can inhibit the activity of the enzyme. Li et al. [57] presented that 1–100 mmol·L^−1^ NH^4+^, Mg^2+^, Ca^2+^, and EDTA could inhibit the activity of the CYP102A16 enzyme in varying degrees, among which Ca^2+^ has the strongest inhibition, and each concentration could inhibit the activity of the CYP102A16 enzyme more than 90%. Plus, acid rain can promote the release of heavy metals in soil, and excessive heavy metals can also inhibit the activity of enzymes [58]. Li et al. [57] showed that the enzyme activity decreased sharply with the increase of metal ion concentration, and in their experiments, 0.2 mmol·L^−1^ Zn^2+^ and 1.0 mmol·L^−1^ Fe^3+^ could completely inactivate the CYP102A16 enzyme. Therefore, multiple factors in the soil have an inhibitory effect on the activity of the CYP3A4 enzyme in the earthworms.

## 4. Conclusions

In the present work, the data suggest that acid rain could cause ecotoxicity to the earthworms, even kill them, which only occurred at extremely strong acid rain (i.e., pH ≤ 2.5). We found that when the acidity of acid rain increased, earthworms gradually showed obvious symptoms of poisoning. Although the earthworms had no obvious pathological symptoms at pH 5.5 of acid rain, the external morphology was still affected. Acid rain could not only cause a stress response of the earthworms but also affect the activity of the CYP3A4 enzyme in the earthworms. It is worth noting that medium-intensity acid rain increased the activity of the CYP3A4 enzyme. The results here further emphasize that the effect of acid rain on the physiological response of earthworms is a complex process.

Nevertheless, field work is lacking in this study to double confirm acid rain influence on earthworms. As this is a preliminary study for the effects of acid rain on the physiological responses of earthworms under simulated conditions, further research in the natural environment is warranted.

## Figures and Tables

**Table 1:** Accumulative mortality of adult *Eisenia fetida* on filter paper under acid rain with eight pH levels for 72 h (*n* = 10). Treatments: pH 7.0: control, pH 2.0–5.5: acid rain with different pH values.

pH	Accumulative Mortality Rate (%)
2.0	100
2.5	40
3.0	0
3.5	0
4.0	0
4.5	0
5.0	0
5.5	0
7.0	0

**Table 2:** Accumulative mortality of adult *Eisenia fetida* in soil under acid rain with eight pH levels for 15 days (*n* = 10). Treatments: pH 7.0: control, pH 2.0–5.5: acid rain with different pH values.

pH	Accumulative Mortality Rate (%)
2.0	100
2.5	20
3.0	0
3.5	0
4.0	0
4.5	0
5.0	0
5.5	0
7.0	0
